# Caffeine and the Risk of Diabetic Retinopathy in Type 2 Diabetes Mellitus: Findings from Clinical and Experimental Studies

**DOI:** 10.3390/nu15051169

**Published:** 2023-02-25

**Authors:** Nuria Alcubierre, Minerva Granado-Casas, Patricia Bogdanov, Cristina Hernández, Hugo Ramos, Esmeralda Castelblanco, Jordi Real, Esther Rubinat-Arnaldo, Alicia Traveset, Marta Hernández, Carmen Jurjo, Jesús Vioque, Eva Maria Navarrete-Muñoz, Rafael Simó, Didac Mauricio

**Affiliations:** 1 Avantmedic Center, 25008 Lleida, Spain; 2 Department of Nursing and Physiotherapy, Health Sciences Faculty, University of Lleida, 25198 Lleida, Spain; 3 Institute of Biomedical Research in Lleida (IRBLleida), 25198 Lleida, Spain; 4 Center for Biomedical Research on Diabetes and Associated Metabolic Diseases (CIBERDEM), Instituto de Salud Carlos III, 08907 Barcelona, Spain; 5 DAP-Cat Group, Unitat de Suport a la Recerca Barcelona, Institut Universitari d’Investigació en Atenció Primària Jordi Gol (IDIAP Jordi Gol), 08041 Barcelona, Spain; 6 Diabetes and Metabolism Research Unit, Vall d’Hebron Research Institute (VHIR) and Autonomous University of Barcelona (UAB), 08035 Barcelona, Spain; 7 Endocrinology, Metabolism and Lipid Research Division, Department of Internal Medicine, Washington University School of Medicine, St. Louis, MO 63110, USA; 8 Department of Ophthalmology, University Hospital Arnau de Vilanova, 25198 Lleida, Spain; 9 Department of Endocrinology & Nutrition, University Hospital Arnau de Vilanova, 25198 Lleida, Spain; 10 Nutritional Epidemiology Unit, Instituto de Investigación Sanitaria y Biomédica de Alicante (ISABIAL), Miguel Hernández University, 46020 Alicante, Spain; 11 CIBER Epidemiología y Salud Pública (CIBERESP), Instituto de Salud Carlos III (ISCIII), 28029 Madrid, Spain; 12 Grupo de Investigación en Terapia Ocupacional (InTeO), Department of Surgery and Pathology, Miguel Hernández University, 03550 Alicante, Spain; 13 Alicante Institute for Health and Biomedical Research (ISABIAL-FISABIO Foundation), 03010 Alicante, Spain; 14 Department of Endocrinology and Nutrition, Hospital de la Santa Creu i Sant Pau & Institut d’Investigació Biomèdica Sant Pau (IIB Sant Pau), 08041 Barcelona, Spain; 15 Faculty of Medicine, University of Vic (UVIC/UCC), 08500 Vic, Spain

**Keywords:** type 2 diabetes, diabetic retinopathy, caffeine intake, coffee consumption, tea consumption, retinal ganglion cell layer

## Abstract

The aim of this study was to assess the potential benefits of caffeine intake in protecting against the development of diabetic retinopathy (DR) in subjects with type 2 diabetes (T2D). Furthermore, we tested the effect of topical administration of caffeine on the early stages of DR in an experimental model of DR. In the cross-sectional study, a total of 144 subjects with DR and 147 individuals without DR were assessed. DR was assessed by an experienced ophthalmologist. A validated food frequency questionnaire (FFQ) was administered. In the experimental model, a total of 20 mice were included. One drop (5 μL) of caffeine (5 mg/mL) (*n* = 10) or vehicle (5 μL PBS, pH 7.4) (*n* = 10) was randomly administered directly onto the superior corneal surface twice daily for two weeks in each eye. Glial activation and retinal vascular permeability were assessed using standard methods. In the cross-sectional study in humans, the adjusted-multivariable model showed that a moderate and high (Q2 and Q4) caffeine intake had a protective effect of DR (odds ratio (95% confidence interval) = 0.35 (0.16–0.78); *p* = 0.011 and 0.35 (0.16–0.77); *p* = 0.010, respectively). In the experimental model, the administration of caffeine did not improve either reactive gliosis or retinal vascular permeability. Our results suggest a dose-dependent protective effect of caffeine in the development of DR, while the potential benefits of antioxidants in coffee and tea should also be considered. Further research is needed to establish the benefits and mechanisms of caffeinated beverages in the development of DR.

## 1. Introduction

Diabetic retinopathy (DR), an ophthalmological complication of diabetes, is the main cause of vision loss and blindness in subjects with diabetes [1]. In Catalonia (Spain), DR is still a relatively frequent microangiopathic complication [2]; furthermore, the incidence of DR is expected to increase due to the increasing incidence of diabetes, obesity, and an ageing population [3]. Nutritional therapy is an integral part of diabetes management and may contribute to the prevention of late diabetes complications [4].

Caffeine (1,3,7-trimethylxanthine) is an active food component with important health implications. The main dietary sources of caffeine intake are coffee, tea, cola or energy drinks, and chocolate, as well as some food products such as gum or alcoholic beverages [5]. Moreover, caffeine is the most consumed behaviorally active compound in the world. The richest food source of caffeine is coffee, used daily by most of the general population worldwide [6]. A meta-analysis performed with prospective studies demonstrated that a higher consumption of coffee is associated with a lower risk of developing type 2 diabetes (T2D) [7]. In contrast, some interventional studies performed with a small sample of subjects with diabetes found that caffeine intake increased postprandial glycaemia (8.9 ± 0.7 mmol/L) compared with baseline (6.7 ± 0.4 mmol/L) after carbohydrate intake [5,8]. A recent prospective study that evaluated the potential association between the consumption of different subtypes of coffee and the incidence of cardiovascular diseases observed a 12% reduction in cardiovascular disease-risk with a consumption of 2–3 cups/day compared with non-drinkers [9]. On the other hand, two reviews performed to assess the beneficial effects of caffeine on different pathologies concluded that the effects in the retina is still uncertain [6,10]. Furthermore, a recent systematic review performed with interventional and observational studies indicated that the association between caffeine and DR is still unclear [11]. A cross-sectional study observed that coffee consumption of over 2 cups/day was inversely correlated with the prevalence of DR in subjects with T2D [12]. However, another cross-sectional study found that daily consumption of caffeine was unfavorable for the retinal microvasculature in adults with increased cardiovascular risk [13], while Neelam et al. [14] did not find any association between coffee consumption and the risk of DR in individuals with diabetes after adjusting for potential confounders. Finally, an in vitro study showed a protective effect of caffeine on the blood retinal barrier in a cellular model of diabetic macular edema, showing an 18% reduction in the apoptosis after caffeine treatment [15].

In terms of the association between tea consumption and the risk of DR, some in vitro-in vivo studies have found a neuroprotective effect of green tea on the retina of diabetic rats [16,17]. Nevertheless, there is a lack of scientific evidence published on this issue.

Overall, inconsistent results about the effect of caffeine intake and DR have been published. To our knowledge, this is the first study to assess the relationship between caffeine intake and DR-risk in subjects with T2D, as well as testing the effect of caffeine in an experimental diabetic model. We hypothesized that caffeine intake could have a protective effect on the development of DR in this population. Therefore, the aim of the study was to assess the potential relationship between caffeine intake (including the consumption of food sources) and DR in subjects with T2D without other late diabetic complications. Furthermore, we tested the effect of topical administration of caffeine on the early stages of diabetic retinopathy in an experimental diabetic model (db/db mouse).

## 2. Materials and Methods

### 2.1. Human Study Design and Subjects

In this cross-sectional study, we included a total of 144 subjects with T2D with DR and 147 individuals with T2D without DR. This is a sub-analysis from a previous published study designed to assess the quality of life and treatment satisfaction of subjects with T2D [17]. All the participants were aged from 40 to 75 years. Participants were recruited from the DR screening and treatment program at the Department of Ophthalmology from March 2010 to January 2013, as reported previously [17]. An experienced ophthalmologist assessed and classified retinopathy according to the international clinical classification system [18]. The exclusion criteria were the presence of other advanced diabetes complications (i.e., macroalbuminuria or renal failure, and the presence of previous cardiovascular disease), and any condition that could affect clinical and nutritional variables (i.e., inflammatory intestinal illness, pancreatitis, chronic hepatic or pulmonary diseases, and pregnancy). The study was conducted according to the guidelines and principles of the Declaration of Helsinki. The local Ethics Committee approved this study, and all the study subjects signed a written informed consent form.

### 2.2. Clinical and Socio-Demographic Characteristics

A detailed description of the assessment of the various clinical variables was recently published [17]. Briefly, the following socio-demographic and clinical characteristics were collected from patient medical records: age, sex, self-reported ethnic group, smoking habit, physical activity, educational level, blood pressure, lipid profile, antihypertensive and lipid-lowering drugs, and glycated hemoglobin (HbA1c). Data on diabetes duration and antidiabetic treatments were also recorded. Blood and urine samples were collected after fasting for 12 h according to standard laboratory methods. Hypertension and dyslipidemia were defined if the subject was being treated with any antihypertensive or lipid-lowering drug, respectively. Physical activity was classified as sedentary if an individual spent less than 10% of daily energy expenditure performing any activity that requires 4 METs (The Metabolic Equivalent) at a minimum (a physical activity with an expenditure similar as walking for 30 min per day), according to the method by Bernstein et al. [19].

### 2.3. Caffeine Intake

The usual intake of foods and nutrients was assessed using a validated 101-item semiquantitative food frequency questionnaire (FFQ) [20]. Briefly, one of the researchers (N.A.) administered the questionnaire to each participant individually. Participants were asked to report how often they had usually consumed each food item over the past year based on nine options ranging from never or less than once a month to 6 or more times per day. Serving sizes were specified for each food item in the FFQ. Nutrient data for each food in the questionnaire were mainly obtained from the US Department of Agriculture’s food composition tables and complemented with Spanish tables of food composition [21,22]. Moreover, nutrient intake and food consumption were adjusted by energy intake using the residual method, where each nutrient is regressed on total calories and the population mean is then added back to the calculated residuals [23]. The caffeine intake was calculated by including the sources of this nutrient as follows: coffee (89.5% of the reported caffeine intake of the study participants), light soft drinks (6.72% of the reported caffeine intake), soft drinks (1.56% of the reported caffeine intake), tea and infusions (0.87%), decaffeinated coffee (0.56%), chocolate (0.59%), chocolate cookies (0.09%), cakes (0.05%), ice cream (0.03%), liquors (0.02%), dairy products (0.01%) and cereals (0.01%).

### 2.4. Experimental Study

Twenty male db/db (BKS.Cg-Dock7m +/+ Leprdb/J) mice and 10 non-diabetic control male mice (db/+) matched by age were purchased from Charles River Laboratories (Calco, Italy). The mice were kept under strict environmental conditions of humidity (60%), cycles of 12h/12 h light/darkness and temperature (20 °C), with unlimited access to filtered water and “ad libitum” food (ENVIGO Global Diet Complete Feed for Rodents, Mucedola, Milan, Italy).

All procedures were performed following the Animal Care and Use Committee of Vall d’Hebron Research Institutem. Furthermore, the study was performed according to the recommendations of the Association for Research in Vision and Ophthalmology Statement for the Use of Animals in Ophthalmic and Vision Research.

Caffeine eye-drops (*n* = 10) or vehicle eye drops (*n* = 10) were randomly administered to 10-week old diabetic mice. Using a micropipette (Eppendorf Research^®^ plus pipette 0.5–10 µL Ref. 3123000098; Hamburg, Germany) one drop (5 μL) of caffeine (5 mg/mL), or vehicle (5 μL phosphate-buffered saline [PBS], pH 7.4) was administered directly onto the superior corneal surface twice per day in each eye for two weeks. Moreover, one drop of both compounds (i.e., caffeine or vehicle) was administered to the eyes 1 h before the mice were euthanized. The investigators who evaluated the results were not aware of the treatment received by the mice.

### 2.5. Assessment of Neurovascular Damage

Glial activation was evaluated in db/db mice treated with caffeine, vehicle and non-diabetic mice (db/+) by analyzing the expression of GFAP (Glial fibrillary acidic protein) using a Laser Scanning Confocal microscope according to the following procedures, as previously reported [24,25]. Paraffin sections were deparaffinized in xylene (VWR, Barcelona, Spain), rehydrated in ethanol (Sigma, St Louis, MO, USA) and fixed in acid methanol (−20 °C) for 1 min. After washing with 0.01 M PBS 4, pH 7.4 (Biowest, Labclinics, Barcelona, Spain), the sections were incubated in blocking solution (3% BSA, Tween 0.05% PBS; Sigma Aldrich, St Louis, MO, USA) for 1 h at room temperature. The sections were incubated with an anti-GFAP rabbit monoclonal (1:500; ab7260; Abcam, Cambridge, UK) overnight at 4 °C, and the following day, were incubated with a fluorescent ALEXA 488 secondary antibody (anti-rabbit or anti-mouse) (Life Technologies S.A, Madrid, Spain) in blocking solution (Protein Block Serum-Free Ready-To-Use DAKO Agilent X0909, Agilent Technologies, Inc., Santa Clara, CA, USA) for 1 h, after washing. Nuclei were counterstained with the blue fluorescent DNA stain Hoechst 33,342 (*2′-[4-ethoxyphenyl]-5-[4-methyl-1-piperazinyl]-2,5′-bi-1H-benzimidazole trihydrochloride trihydrate*) (Thermo Fisher Scientific, OR, USA) after washing. Last, the sections were mounted in Prolong Gold antifade mounting medium (Invitrogen, Thermo Fisher Scientific, Bend, OR, USA) using a coverslip. Images were captured with a confocal laser scanning microscope (FV1000; Olympus, Hamburg, Germany) at a resolution of 1024 × 1024 pixels. Five fields (three from the central retina and two from the peripheral retina) from each section were analyzed using ImageJ software (National Institutes of Health, Bethesda, MD, USA). The degree of glial activation as determined by the GFAP staining was scored as follows: Müller cell end-feet region/ganglion cell layer (GCL) only (score 1); Müller cell end-feet region/GCL plus a few proximal processes (score 2); Müller cell end-feet plus many processes, but not extending to the inner nuclear layer (INL) (score 3); Müller cell end-feet plus processes throughout with some in the outer nuclear layer (ONL) (score 4); Müller cell end-feet plus many dark processes from the GCL to the outer margin of the ONL (score 5) [26].

The permeability of retinal vascular was assessed by measuring the leakage of albumin from the blood vessels into the retina using the well-established Evans Blue albumin method (ex vivo). Briefly, Evans Blue (E2129 SIGMA, Sant Louis, MO, USA) was injected intraperitoneally (17 mg/kg body weight, at a concentration of 5 mg/mL dissolved in PBS pH 7.4), following which the mice turned visibly blue, demonstrating the correct uptake and distribution of the dye. The mice were euthanized after 120 min, the eyes were enucleated and flat-mounted slides were prepared and cover slipped with a drop of Prolong Gold antifade mounting medium (Invitrogen, Thermo Fisher Scientific, Waltham, MA, USA). A confocal laser scanning microscope (FV1000; Olympus, Hamburg, Germany) was used to generate digital images from a number of random fields from all the retinas at 60× using a 561-nm laser line, with each image being recorded with an identical beam intensity at a resolution of 1024 pixels × 1024 pixels. Z-stack retinal images (step size 1.16 μm) of different regions of the vascular tree were acquired and the number of extravasations per field (at 60×) was counted to analyze the albumin-bound Evans Blue.

### 2.6. Statistical Analysis

Mean values (with standard deviations [SD]) or absolute and relative frequencies (in percentages) were derived for quantitative or qualitative variables, respectively. Mann-Whitney tests or Chi-squared tests were used to compare whether there were differences between the groups. Bonferroni-corrected *p*-values were also provided to adjust for multiple pairwise comparisons. Bivariable analysis between caffeine intake, coffee and tea consumption and the outcome, i.e., the presence of DR, was performed to obtain crude odds ratio (OR) and their 95% confidence interval (95% CI). Multiple logistic regression was used to explore the association between independent variables (i.e., caffeine intake, coffee, and tea consumption) and the outcome variable (DR). We estimated OR and their 95% CI, adjusting for potential confounding factors such as age, sex, educational level, physical activity, hypertension, dyslipidemia, diabetes duration, glycated hemoglobin (HbA1c), body mass index (BMI) and tobacco exposure.

In the animal experiments, statistical comparisons were performed using a one-way ANOVA performing the Bonferroni test. For statistical purposes, the GFAP score (extent of glial activation) was categorized as “normal” (scores 1 and 2) and “pathological” (scores 3, 4 and 5), and any differences between groups was analyzed using the Fisher’s test.

For all comparisons, the significance level was set at 0.05. The data was analyzed using R 3.0.1 software [27].

## 3. Results

### 3.1. Clinical Study

The clinical characteristics of the study participants are shown in Table 1. In comparison with individuals with no DR, those with this condition were older (*p* = 0.024) and showed a larger waist circumference (*p* = 0.034), higher systolic blood pressure (SBP) (*p* < 0.001), a higher frequency of hypertension (*p* = 0.005), longer diabetes duration (*p* < 0.001), higher glycated hemoglobin (HbA1c) levels (*p* < 0.001), and higher high density lipoprotein cholesterol (HDL-c) levels (*p* = 0.031). In addition, these subjects had a lower educational level (*p* = 0.003). In terms of diabetes treatment, a higher frequency of subjects with DR were treated with both oral antidiabetic drugs (OAD) and insulin (*p* < 0.001).

#### Caffeine Intake and Diabetic Retinopathy

We observed a higher frequency of subjects with DR in the lowest quartile of caffeine intake compared with individuals without DR (30.6% vs. 19.7%, *p* = 0.045) (Table 2). However, we did not observe differences between groups in coffee and tea consumption. On the other hand, the unadjusted bivariable analysis showed a protective association between the Q2 and Q4 quartiles of caffeine intake and the prevalence of DR (odds ratio [OR] (95% confidence interval [CI]) = 0.46 (0.24–0.90); *p* = 0.022 and 0.47 (0.24–0.92); *p* = 0.027, respectively) (Table 2). However, no significant association was observed between coffee and tea consumption, and the prevalence of DR.

The multivariable logistic models performed with caffeine intake and coffee and tea consumption as continuous variables did not show any association between caffeine intake or coffee and tea consumption and the presence of DR (Table 3). However, hypertension and diabetes duration were associated with a high risk of DR in both models (OR (95% CI) = 1.91 (1.04–3.52); *p* = 0.037 and 1.87 (1.02–3.46); *p* = 0.043 for hypertension) and (OR (95% CI) = 1.10 (1.06–1.15); *p* < 0.001 and 1.10 (1.06–1.16); *p* < 0.001 for diabetes duration), respectively.

On the other hand, the multivariable model performed with quartiles of caffeine intake showed that a moderate and high (Q2 and Q4) daily intake of caffeine had a protective effect on DR (OR (95% CI) = 0.35 (0.16–0.78); *p* = 0.011 and 0.35 (0.16–0.77); *p* = 0.010, respectively) (Table 4). However, we did not observe any association between quartiles of coffee and tea consumption and the prevalence of DR. As expected, hypertension (OR (95% CI) = 2.16 (1.17–4.05), *p* = 0.014 and 1.87 (1.02–3.47), *p* = 0.043), diabetes duration (OR (95% CI) = 1.11 (1.06–1.16), *p* < 0.001 and 1.10 (1.06–1.16), *p* < 0.001) and HbA1c (OR (95% CI) = 1.60 (1.28–2.03), *p* < 0.001 and 1.55 (1.24–1.97), *p* < 0.001) were identified as risk factors of DR in both models.

### 3.2. Experimental Study

We did not find any differences in blood glucose concentrations and body weight during the study between db/db mice treated with caffeine and db/db mice treated with vehicle.

**Figure 1:**
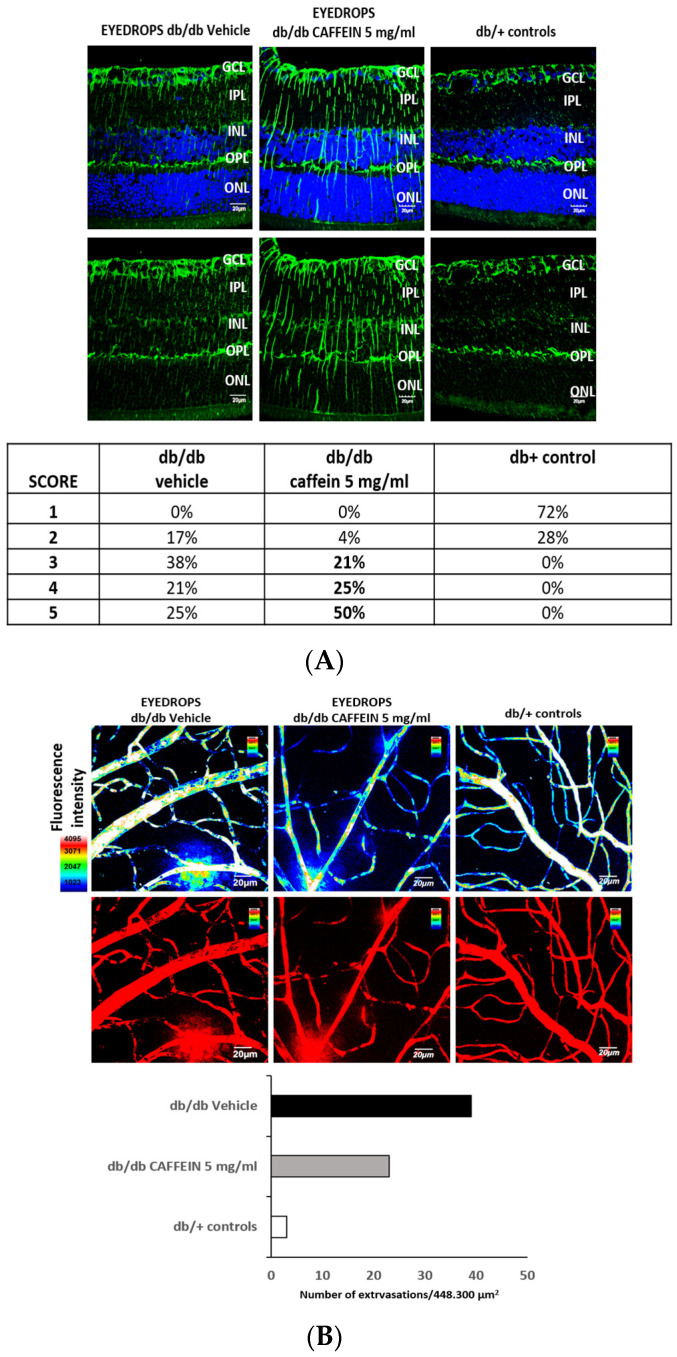
(**A**) The effect of caffeine eye drops on glial activation. Upper panel: Comparison of GFAP immunoreactivity (green) in the retina among representative samples from a diabetic mouse treated with vehicle, a diabetic mouse treated with caffeine, and a non-diabetic mouse. Nuclei were labeled with Hoechst (blue). ONL: outer nuclear layer; OPL: outer plexiform layer; INL: inner nuclear layer; IPL: inner plexiform layer; GCL: ganglion cell layer. Scale bars, 20 µm. Lower panel: Quantification of glial activation based on the extent of GFAP staining. N: 6 mice per group; (**B**) Effect of caffeine eye drops on vascular leakage. Upper panel: Confocal immunofluorescence images of vascular permeability assessed by Evans Blue dye leakage in retinal whole mounts. Spec3, fluorescent spectral signature 3. FI: fluorescence intensity. Scale bars, 20 μm. Lower panel: For quantification, the number of extravasations per field of 60× retina was counted. N: 4 mice per group.

As expected, in non-diabetic control mice (db/+) GFAP expression was mainly confined to the retinal ganglion cell layer (GCL) (Figure 1A). The diabetic mice (db/db) treated with vehicle presented with significantly higher GFAP expression than non-diabetic mice matched by age (Fisher’s exact test: *p* < 0.01). The administration of caffeine was not able to decrease reactive gliosis.

The number of vascular extravasations was significantly reduced in non-diabetic mice compared with diabetic mice treated with vehicle. A lower rate of vascular extravasations was observed in diabetic mice treated with caffeine eye drops in comparison with those treated with vehicle, but the difference was not statistically significant (Figure 1B).

## 4. Discussion

Our results suggest a protective effect of moderate and high (Q2 and Q4) daily caffeine intake and the presence of DR. However, no association was found between coffee and tea consumption, the main food sources of caffeine, and the presence of DR in these individuals. In addition, when we performed the experimental study with db/db mice, we did not observe any effect of caffeine on the retina.

In our study, a protective association between moderate and high (Q2 and Q4) caffeine intake and the risk of DR was found; however, this relationship was not observed when the analysis was performed with caffeine intake as a continuous variable, and there was no relationship between DR and daily coffee and tea consumption, suggesting a non-linear association between caffeine and DR. In scientific literature, conflicting results have been published. A cross-sectional study performed in Korea with a large sample of subjects with T2D found an inverse correlation between the consumption of 2 or more cups of coffee per day and the prevalence of any type of DR after adjusting for potential confounders, which is in line with our results [12]. On the other hand, another cross-sectional study performed with a small sample of subjects with the presence of one or more cardiovascular risk factors, including diabetes, found that caffeine intake was positively associated with retinal venular caliber suggesting that caffeine might have an unfavorable effect on the retinal microvasculature, which is in contrast to our results [13]. Nevertheless, the authors did not observe any association between coffee and tea consumption and retinal vessel calibers. Furthermore, two interventional studies performed with small samples of healthy subjects found that caffeine intake (100 mg in one study and 200 mg in the other study) had an acute constricting effect in the retina [28,29]; this is also in contrast with our results. Nevertheless, a cross-sectional study performed with a large sample of subjects with diabetes did not find any association between coffee consumption and the risk of macular degeneration and DR after adjusting for potential confounders [14]. As described in a review, epidemiological studies have shown a preventive effect of green tea in the development of DR [30], showing a risk-reduction in 50% compared with non-drinkers [31]. However, no interventional studies have been performed to assess the effects of tea consumption on the prevention of DR [30]. A recent systematic review of interventional and observational studies concluded that the association between the consumption of coffee and DR is still unclear [11]; therefore, further studies are needed to establish the effect of caffeine intake on DR.

We did not observe any effect of caffeine in the retina in our in vitro study in db/db mice. This is in contrast with an in vitro study performed with human cells that found a protective effect of caffeine that was shown to inhibit apoptotic cell death induced by hyperglycemic/hypoxic insult [15]. Furthermore, a recent narrative review described decreased ischemic injury after caffeine exposure in animal models of glaucoma [32]. Moreover, a possible protective effect of green tea reducing the production of reactive oxygen species in the retinal nerves of diabetic rats was previously described [16,30]. The possible mechanisms behind the neuroprotective effects of caffeine have been related to the antagonism of adenosine receptors in retinal degenerative diseases [6]. Neural damage mainly occurs because of energy deficit and ionic imbalance, thus leading to oxidative stress and inflammation in the retina. Moreover, an increased adenosine concentration after an ischemic event should be considered due to the protective benefits of adenosine receptors [32].

Coffee and tea are major contributors of total antioxidant intake, as chlorogenic acid and flavonoids, respectively, which could have protective effects on the development of certain forms of cancer, arthritis and cardiovascular diseases [6,16]. An in vitro study showed that coffee extract and chlorogenic acid reduced the apoptosis of retinal cells induced by hypoxia and nitric oxide, suggesting a prevention of coffee consumption in retinal degeneration [33]. In addition, an in vivo–in vitro study showed a reduction in vascular retinal damage with chlorogenic acid [34]. Moreover, a recent review described the potential protective effect of chlorogenic acid for the prevention of diabetic complications [35]; however, there are still a lack of robust clinical trials assessing the application of chlorogenic acid. In our experimental study, caffeine had no effect on the retina. Moreover, in the clinical study, no association was found between the consumption of tea and coffee drinks and DR. It is, therefore, possible that other compounds contained in caffeinated beverages such as antioxidants may be responsible for the beneficial effects.

The limitations of this study are the cross-sectional design of the clinical study whereby a causal relationship between caffeine intake and the development of DR cannot be established. Moreover, this is a sub-analysis of a previous study, mainly designed to assess the quality of life and treatment satisfaction of subjects with T2D [17]. Furthermore, the small sample size might have reduced the probability of finding statistical significance. In addition, the presence of other compounds in caffeinated beverages, i.e., antioxidants, that were not analyzed could influence the results of the study. On the other hand, this study was performed with a large and well-defined sample of individuals with DR and non-DR without other late diabetic complications. The FFQ can be used to estimate caffeine intake and food consumption over the previous five-year period showing good reproducibility [20,36]. Moreover, this is the first study that assessed the relationship between caffeine intake, including coffee and tea consumption, and the development of DR, encompassing both clinical and experimental aspects.

## 5. Conclusions

A moderate and high caffeine intake was associated with a 65% reduced risk of DR in subjects with T2D without other late diabetic complications. However, the experimental model did not support the findings in humans. This could be due to the presence of other potential compounds, i.e., antioxidants, in coffee and tea which could have protective effects in the retina. Nevertheless, further studies are needed to determine the possible benefits and effects of caffeine intake and other compounds which are present in coffee and tea, and the potential mechanisms related to the prevention of DR and retinal damage in subjects with diabetes mellitus.

## Figures and Tables

**Table 1:** Clinical characteristics of the study subjects.

Characteristics	No DR (*n* = 147)	DR (*n* = 144)	*p*
Age (years)	57.9 (10.3)	60.4 (8.8)	0.024
Sex (women)	71 (48.3%)	72 (50.0%)	0.863
Educational level			0.003
No university	130 (88.4%)	141 (97.9%)	
Graduate or higher	17 (11.6%)	3 (2.1%)	
Tobacco exposure	81 (55.1%)	71 (49.3%)	0.350
Physical activity			0.855
Sedentary	75 (51.0%)	76 (52.8%)	
Active	72 (49.0%)	68 (47.2%)	
BMI (Kg/m^2^)	31.3 (5.1)	31.9 (5.6)	0.358
Waist (cm)	104.0 (12.1)	107.0 (11.1)	0.034
SBP (mmHg)	134.0 (15.5)	145.0 (19.9)	<0.001
DBP (mmHg)	76.6 (10.5)	77.0 (11.1)	0.723
Hypertension	73 (49.7%)	96 (66.7%)	0.005
Dyslipidemia	64 (43.5%)	74 (51.4%)	0.221
Diabetes duration (years)	7.07 (5.5)	14.0 (9.9)	<0.001
Diabetes treatment			<0.001
Insulin	4 (2.7%)	17 (11.8%)	
OAD	94 (63.9%)	64 (44.4%)	
OAD + insulin	13 (8.8%)	60 (41.7%)	
Diet	36 (24.5%)	3 (2.1%)	
HbA1c (%)	7.3 (1.2)	8.2 (1.4)	<0.001
HbA1c (mmol/mol)	55.9 (12.7)	66.6 (15.6)	<0.001
Total cholesterol (mg/dL)	186.0 (36.7)	185 (36.2)	0.790
HDL-c (mg/dL)	48.5 (10.8)	51.8 (14.8)	0.031
LDL-c (mg/dL)	111.0 (30.8)	106.0 (30.3)	0.177
Triglycerides (mg/dL)	138.0 (82.1)	141.0 (121.0)	0.827

Data are shown as mean (SD) for continuous variables and *n* (%) for categorical variables. BMI, body mass index; DBP, diastolic blood pressure; DR, diabetic retinopathy; HbA1c, glycated hemoglobin; HDL-c, high density lipoprotein cholesterol; LDL-c, low density lipoprotein cholesterol; OAD, oral antidiabetic drugs; SBP, systolic blood pressure. Tobacco exposure includes current and former smokers.

**Table 2:** Bivariable analysis of caffeine intake, coffee and tea consumption and the presence of diabetic retinopathy.

Variables	No DR (*n* = 147)	DR (*n* = 144)	p.overall ^1^	OR (95% CI)	*p* ^2^
Energy intake (kcal/day)	2200.0 (582.0)	2090.0 (584.0)	0.122	-	-
Caffeine (g/day)	1.9 (2.3)	1.6 (2.0)	0.217	0.93 (0.84–1.04)	0.218
Caffeine (g/day)			0.045		
Q1 (0.00–0.10)	29 (19.7%)	44 (30.6%)		Ref.	Ref.
Q2 (0.11–1.08)	43 (29.3%)	30 (20.8%)		0.46 (0.24–0.90)	0.022
Q3 (1.09–3.10)	33 (22.4%)	40 (27.8%)		0.80 (0.41–1.55)	0.509
Q4 (3.11–9.55)	42 (28.6%)	30 (20.8%)		0.47 (0.24–0.92)	0.027
Coffee and tea (g/day)	3.6 (3.3)	3.0 (2.9)	0.105	0.94 (0.87–1.01)	0.110
Coffee and tea (g/day)			0.376		
Q1 (0.00–0.95)	30 (20.4%)	42 (29.2%)		Ref.	Ref.
Q2 (0.96–2.09)	38 (25.9%)	35 (24.3%)		0.66 (0.34–1.27)	0.216
Q3 (2.10–5.02)	40 (27.2%)	33 (22.9%)		0.59 (0.30–1.14)	0.118
Q4 (5.03–26.06)	39 (26.5%)	34 (23.6%)		0.63 (0.32–1.21)	0.162

Caffeine intake, coffee and tea consumption were calculated by adjusting for energy intake. ^1^ p.overall for the comparison between both groups. ^2^ *p* value for the bivariable analysis between each variable and the presence of diabetic retinopathy. Q1–Q4 are quartiles of consumption corresponding to low, moderate, high and very high. CI, confidence interval; DR, diabetic retinopathy; OR, odds ratio.

**Table 3:** Multivariable logistic models for the association between the caffeine intake and coffee and tea consumption with the presence of diabetic retinopathy.

	Diabetic Retinopathy			
Variables	OR (95% CI) ^1^	*p*	OR (95% CI) ^2^	*p*
(Intercept)	0.00 (0.00–0.10)	0.001	0.00 (0.00–0.12)	0.002
Caffeine (g/day)	0.93 (0.81–1.05)	0.241	-	-
Coffee and tea (g/day)	-	-	0.91 (0.82–1.01)	0.087
Age (years)	1.00 (0.97–1.03)	0.833	1.00 (0.96–1.03)	0.779
Sex (women)	0.58 (0.28–1.17)	0.133	0.55 (0.27–1.11)	0.098
Educational level (no university)	2.93 (0.84–13.77)	0.121	2.79 (0.80–13.13)	0.139
Physical activity (active)	0.91 (0.52–1.59)	0.744	0.90 (0.51–1.56)	0.702
Hypertension	1.91 (1.04–3.52)	0.037	1.87 (1.02–3.46)	0.043
Dyslipidemia	1.03 (0.59–1.79)	0.922	1.05 (0.60–1.84)	0.855
Diabetes duration (years)	1.10 (1.06–1.15)	<0.001	1.10 (1.06–1.16)	<0.001
BMI (Kg/m^2^)	1.02 (0.96–1.08)	0.584	1.02 (0.96–1.08)	0.583
HbA1c (%)	1.56 (1.25–1.97)	<0.001	1.57 (1.26–1.99)	<0.001
Tobacco exposure	0.96 (0.49–1.90)	0.917	1.02 (0.51–2.03)	0.957

Caffeine intake, coffee and tea consumption were calculated adjusting for energy intake. ^1^ Multivariable logistic model for the relationship between caffeine intake and DR. ^2^ Multivariable logistic model for the association between coffee and tea consumption and DR. BMI, body mass index; CI, confidence interval; HbA1c, glycated hemoglobin; OR odds ratio. Tobacco exposure includes current and former smokers.

**Table 4:** Multivariable logistic models for the association between the quartiles of caffeine, coffee and tea consumption and the presence of diabetic retinopathy.

	Diabetic Retinopathy			
Variables	OR (95% CI) ^1^	*p*	OR (95% CI) ^2^	*p*
(Intercept)	0.01 (0.00–0.19)	0.004	0.00 (0.00–0.12)	0.002
Caffeine (g/day)				
Q1 (0.00–0.10)	Ref.	Ref.	-	-
Q2 (0.11–1.08)	0.35 (0.16–0.78)	0.011	-	-
Q3 (1.09–3.10)	0.88 (0.40–1.91)	0.741	-	-
Q4 (3.11–9.55]	0.35 (0.16–0.77)	0.010	-	-
Coffee and tea (g/day)				
Q1 (0.00–0.95)	-	-	Ref.	Ref.
Q2 (0.96–2.09)	-	-	0.56 (0.25–1.22)	0.145
Q3 (2.10–5.02)	-	-	0.62 (0.28–1.34)	0.225
Q4 (5.03–26.06)	-	-	0.53 (0.24–1.18)	0.123
Age (years)	0.99 (0.96–1.02)	0.585	1.00 (0.97–1.03)	0.988
Sex (women)	0.55 (0.26–1.13)	0.110	0.56 (0.27–1.13)	0.108
Educational level (no university)	2.94 (0.80–14.41)	0.132	3.05 (0.87–14.45)	0.109
Physical activity (active)	0.87 (0.49–1.53)	0.629	0.92 (0.52–1.61)	0.763
Hypertension	2.16 (1.17–4.05)	0.014	1.87 (1.02–3.47)	0.043
Dyslipidemia	0.93 (0.53–1.65)	0.815	1.03 (0.59–1.80)	0.915
Diabetes duration (years)	1.11 (1.06–1.16)	<0.001	1.10 (1.06–1.16)	<0.001
BMI (Kg/m^2^)	1.01 (0.96–1.07)	0.655	1.01 (0.96–1.08)	0.617
HbA1c (%)	1.60 (1.28–2.03)	<0.001	1.55 (1.24–1.97)	<0.001
Tobacco exposure	0.98 (0.49–1.98)	0.963	0.99 (0.50–1.97)	0.985

Caffeine intake, coffee and tea consumption were calculated adjusting by energy intake. Q1–Q4 are quartiles of consumption. ^1^ Multivariable logistic model for the relationship between quartiles of caffeine intake and DR. ^2^ Multivariable logistic model for the association between quartiles of coffee and tea consumption and DR. BMI, body mass index; CI, confidence interval; HbA1c, glycated hemoglobin; OR, odds ratio. Tobacco exposure includes current and former smokers.

## Data Availability

The data presented in this study are available on request from the corresponding author.
